# Coinfection of *Clonorchis sinensis* and hepatitis B virus: clinical liver indices and interaction in hepatic cell models

**DOI:** 10.1186/s13071-022-05548-5

**Published:** 2022-12-12

**Authors:** Huimin Dong, Lu Zhao, Hengchang Sun, Mei Shang, Gang Lv, Xinbing Yu, Bo Hu, Yan Huang

**Affiliations:** 1 Department of Laboratory Medicine, The Third Affiliated Hospital of Sun Yat-Sen University, Guangzhou, Guangdong People’s Republic of China; 2 Department of Parasitology, Zhongshan School of Medicine, Sun Yat-Sen University, Guangzhou, Guangdong People’s Republic of China; 3 Key Laboratory for Tropical Diseases Control of Ministry of Education, Sun Yat-Sen University, Guangzhou, Guangdong People’s Republic of China; 4 Provincial Engineering Technology Research Center for Biological Vector Control, Guangzhou, Guangdong People’s Republic of China; 5 Department of Clinical Laboratory, The Sixth Affiliated Hospital of Sun Yat-Sen University, Guangzhou, Guangdong People’s Republic of China; 6 Key Laboratory of Tropical Translational Medicine of Ministry of Education, Hainan Medical University, Haikou, Hainan People’s Republic of China

**Keywords:** *Clonorchis sinensis*, Hepatitis B virus, Total proteins from *C. sinensis* adults (*Cs*TPs), Coinfection, Liver fibrosis

## Abstract

**Background:**

In China, people infected with hepatitis B virus (HBV) are commonly found in areas with a high prevalence of *Clonorchis sinensis*, a trematode worm. Published studies have reported that the progression of hepatitis B is affected by coinfection *C. sinensis*.

**Methods:**

Clinical data from a total of 72 patients with *C. sinensis* and HBV (as sole infection or with coinfections) and 29 healthy individuals were analysed. We also incubated the hepatic stellate cell line LX-2 with total proteins from *C. sinensis* adult worms (*Cs*TPs) and HBV-positive sera. In addition, the human hepatoblastoma cell line HepG2.2.15 was treated with the antiviral drug entecavir (ETV), *Cs*TPs and the anti-*C. sinensis* drug praziquantel (PZQ).

**Results:**

Our clinical data indicated that the levels of alanine aminotransferase (ALT), aspartate aminotransferase (AST), total bilirubin (TB) and hyaluronic acid (HA) were significantly higher in patients with coinfection than in those infected with HBV only. In cell models, compared with the model in which LX-2 cells were incubated with HBV-positive sera (HBV group), transcripts of alpha-smooth muscle actin and types I and III collagen were significantly elevated in the models of LX-2 cells treated with *Cs*TPs and HBV-positive sera (*Cs*TP+HBV group), while the messenger RNA levels of tumour necrosis factor-α, interleukin (IL)-1β and IL-6 in the *Cs*TP+HBV group were clearly lower. The HBV surface antigen and hepatitis B e-antigen levels were higher in the HepG2.2.15 cells treated with ETV and *Cs*TPs than in those in the ETV group and in the cells administered a mixture of ETV, *Cs*TPs and PZQ.

**Conclusions:**

These results confirmed that *C. sinensis* and HBV coinfection could aggravate the progression of liver fibrosis. *Cs*TPs might promote chronic inflammation of the liver in individuals with HBV infection, resulting in the development of hepatic fibrosis.

**Graphic abstract:**

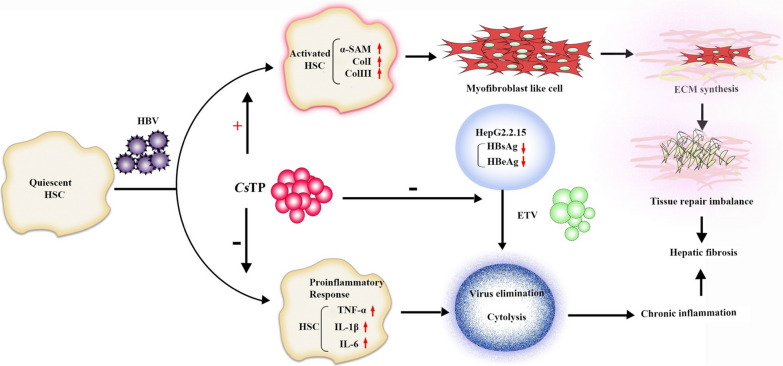

**Supplementary Information:**

The online version contains supplementary material available at 10.1186/s13071-022-05548-5.

## Background

Adult *Clonorchis sinensis* worms inhabit the intrahepatic bile ducts of humans, causing the food-borne parasitic disease clonorchiasis [1]. It has been reported that approximately 15 million people are infected with *C. sinensis* globally, primarily in China, South Korea, northern Vietnam and the far east of Russia, with approximately 13 million of these cases in China, especially in Guangdong Province and the Guangxi Zhuang Autonomous Region of South China [2–4].

Hepatitis B virus (HBV) attacks liver cells, leading to hepatitis B. The WHO estimated that 296 million people were living with chronic hepatitis B in 2019, with 1.5 million new infections occurring each year. Approximately 15–25% of people with chronic HBV infection die from liver cirrhosis or hepatocellular carcinoma [5]; in 2019, an estimated 820,000 people died from HBV-related illnesses, presenting a serious public health problem. China belongs to the countries classified as medium prevalence areas, with an HBV surface antigen (HBsAg)-positive rate of 6.89% [6].

In China, people infected with HBV are commonly found in areas with a high prevalence of *C. sinensis* [7, 8]. The HBsAg-positive rate has been documented to be significantly higher in endemic areas of *C. sinensis* than in nonendemic areas [9, 10]. It is generally accepted that dysfunctional immune responses play an essential role in persistent HBV infection, as well as liver inflammation and hepatic fibrosis [11]. Adult parasites can trigger type 2 or mixed type 1/type 2 immune responses in the host [12]. *Clonorchis sinensis* infection also induces the activation of CD4^+^ lymphocytes, including T helper 1/2/17 (Th1/Th2/Th17) cells and regulatory T (Treg) cells, in FVB mice [13]. In our previous study, we found that patients coinfected with *C. sinensis* and HBV showed higher serum interleukin (IL)-6 and IL-10 levels and lower serum interferon gamma (IFN-γ) levels, suggesting that coinfection could exacerbate the imbalance of Th1/Th2 cytokines [14]. These results also indicated that *C. sinensis* coinfection might regulate immune responses and then affect the progression of hepatitis B as well as liver fibrosis.

In the current study, we investigated further the effects of coinfection on liver fibrosis by analysing clinical liver indices and using a cell model to detect the levels of fibrosis-related molecules and cytokines. Previously, we also found that coinfected patients presented weaker liver function and higher numbers of HBV DNA copies. In addition, combination treatment of coinfected patients with antiviral (entecavir [ETV]) and anti-*C. sinensis* drugs (praziquantel [PZQ]) could contribute to a reduction in viral load and facilitate the recovery of liver function. In the present study, we also analysed the effect of ETV and/or PZQ on a cell model to confirm the above effects.

## Methods

### Study population

The study population comprised 72 patients with HBV, *C. sinensis* or both infections diagnosed at the Third Affiliated Hospital of Sun Yat-sen University and 29 healthy individuals serving as healthy controls (HCs), who were enrolled in parallel. The enrolled men and women were aged ≥ 18 years. The participants were divided into four groups: (i) the coinfection group (*n* = 22), including patients who were positive for both *C. sinensis* eggs and HBsAg with HBV DNA > 20 IU/ml; (ii) the HBV group (*n* = 26), including patients who were HBsAg-positive with HBV DNA > 20 IU/ml; (iii) the *C. sinensis* group (*n* = 24), including patients who were positive for *C. sinensis* eggs only; and (iv) the HC group, including individuals matched for age and sex (*n* = 29). Patients with the following conditions were excluded: coinfection with hepatitis A, C, D or E, human immunodeficiency virus, *Schistosoma japonicum*, *Schistosoma mansoni* or other parasites; hepatitis B cirrhosis; alcoholic liver disease; autoimmune diseases; serious heart diseases; malignant tumours; cholestasis; diabetes. Pregnant women were also excluded.

### Biochemical and serological testing

The levels of hyaluronic acid (HA), type III procollagen (PCIII), type IV collagen (COLIV) and laminin (LN) in sera were measured using a BL-9600 Micropore Plate Chemiluminescent Immunity Analyser (Bell Medical Electronics Ltd., Tianjin, China). The presence of HBV DNA was tested by real-time PCR with a lower detection limit of 20 IU/ml (Roche Diagnostics, Mannheim, Germany). Serum HBsAg and hepatitis B e-antigen (HBeAg) were detected using an electrochemiluminescence immunoassay (ECLI) kit for the COBAS-e801 system (Roche Diagnostics). The enzymatic activity levels of alanine aminotransferase (ALT), aspartate aminotransferase (AST) and total bilirubin (TB) were tested with automated biochemical techniques (Hitachi 7600 automatic biochemical analyser; Hitachi Ltd., Tokyo, Japan).

### Collection of total proteins from *C. sinensis* adults before and after treatment with PZQ

Freshly collected adult worms of *C. sinensis* were gently rinsed several times with phosphate-buffered saline (PBS, pH 7.2) supplemented with penicillin and streptomycin (100 U/ml and 100 μg/ml, respectively; Gibco Life Technologies, Thermo Fisher Scientific, Waltham, MA, USA) and identified using previously reported protocols [15]. The worms were then lysed in 1 ml of PBS at an oscillation frequency of 30 Hz for 10 min. The supernatant containing total proteins extracted from *C. sinensis* adults (*Cs*TPs) was harvested and centrifuged at 4000 rpm for 15 min at 4 °C. *Cs*TPs were filtered through a sterile 0.22-μm syringe filter and stored at − 80 °C after quantification until use [16]. The protein concentration was detected using a BCA Protein Assay Kit (Thermo Fisher Scientific).

PZQ is a pyrazinoquinoline compound that is effective for treating patients with trematode infections, including *C. sinensis*, and is commonly used to treat patients with such infections. It has been proposed that this compound may affect the tegument and musculature of the worm. In this study, 1 mg of PZQ (GKH Pharmaceutical, Ltd., Guangzhou, China) was dissolved in 1 ml of PBS containing 60% dimethyl sulfoxide and then diluted with PBS to final concentrations of 0.1, 1 and 10 μg/ml, respectively [17, 18]. The PZQ solutions were successively filtered through a sterile 0.22-μm syringe filter. Ten active *C. sinensis* adult worms that had been cleaned were treated with the different concentrations of PZQ in Dulbecco's Modified Eagle Medium (DMEM; Gibco Life Technologies, Thermo Fisher Scientific) in vitro for 12, 24 or 48 h. *Cs*TPs from the treated adults were collected using the methods mentioned above.

### Coeffects of *Cs*TPs and HBV on human hepatic stellate cell line LX-2

LX-2 cells (1 × 10^4^ cells/ml) were incubated with different concentrations of *Cs*TPs (0, 20, 40, 80 μg/ml) or HBV-positive sera with different numbers of HBV DNA copies (0, 1.0 × 10^8^, 2.0 × 10^8^ or 4.0 × 10^8^ HBV DNA IU/ml) for 24 h in a round-bottomed 96-well plate (Costar®; Corning Ltd., Corning, NY, USA). LX-2 cells in 0.5 μg/ml bovine serum albumin (BSA; MBCHEM, Shanghai, China) in RPMI 1640 medium (Gibco Life Technologies, Thermo Fisher Scientific) were used as blank controls, and LX-2 cells in RPMI 1640 medium with HBV-negative sera from healthy individuals were used as negative controls. Cell proliferation was assessed by measuring optical density at 450 nm (OD_450_) using the Cell Counting Kit 8 Assay (CCK-8 assay; Dojindo Laboratories, Kumamoto, Japan) to determine the optimal concentration of *Cs*TPs or HBV DNA copies with the strongest effects.

An aliquot of 2.0 × 10^5^/ml viable LX-2 cells in RPMI 1640 medium was seeded into 6-well cell culture clusters and incubated for 24 h at 37 °C under 5% CO_2_ in an incubator with HBV-positive sera (2.0 × 10^8^ HBV DNA IU/ml), *Cs*TPs (40 μg/ml) or both (*Cs*TP+HBV group). LX-2 cells in RPMI 1640 medium treated with HBV-positive sera (2.0 × 10^8^ HBV DNA IU/ml) and 0.5 μg/ml BSA) were used as a control group (BSA+HBV group). Cells incubated with PBS or HBV-negative sera in RPMI 1640 medium were used as a blank control. Total RNA was extracted from LX-2 cells from each group using TRIzol reagent (TransGen Biotech Co. Ltd., Beijing, China). Complementary DNAs (cDNAs) were then synthesized using a TransScript All-in-One First-Strand cDNA Synthesis SuperMix for qPCR (One-Step gDNA Removal Kit; TransGen Biotech Co. Ltd.) from 1 μg of total RNA according to the manufacturer’s protocol. Quantitative real-time PCR (Q-PCR) was performed on a CFX96 Real-Time PCR Detection System (Bio-Rad Laboratories, Hercules, CA, USA) using TransStart Top/Tip Green qPCR SuperMix (TransGen Biotech Co. Ltd.). The PCR cycling conditions were: a denaturation step at 94 °C for 30 s, followed by 40 cycles of 94 °C for 5 s, 60 °C for 30 s, 95 °C for 5 s, with a final cycle at 60 °C for 10 s. The messenger RNA (mRNA) levels of alpha-smooth muscle actin (α-SMA), types I and III collagen, tumour necrosis factor alpha (TNF-α), IL-1β and IL-6 were analysed by calculating 2^−ΔΔCt^ and normalizing to β-actin mRNA. All primers for Q-PCR are shown in Additional file 1: Table S1. All assays were performed in triplicate and repeated twice.

### HBsAg, HBeAg and HBV DNA copies of the treated hepatoma cell line HepG2.2.15

The HepG2.2.15 cell line is characterized by stable HBV expression and replication in the culture system. It is an effective model for screening for anti-HBV drugs in vitro and studying the structure, function, gene expression and regulation of HBV DNA [19]. Moreover, it has been documented that anti-helminth drugs such as albendazole could affect the enzymatic activity of excretory-secretory products of cestodes [20]. In this study, HepG2.2.15 cells were employed to analyse the effects of ETV with or without *Cs*TPs pretreated with PZQ on HBV.

A total of 5.0 × 10^4^ viable HepG2.2.15 cells seeded into 12-well plates containing 200 μl of DMEM were treated with one of the following treatments: (i) 30 nmol/ml ETV (Selleck Chemicals, Houston, TX, USA); (ii) 30 nmol/ml ETV and 40 μg/ml *Cs*TPs (ETV+*Cs*TPs); (iii) 40 μg/ml *Cs*TPs; (iv) 0.5 μg/ml BSA and 30 nmol/ml ETV (ETV+BSA); or (v) ETV (30 nmol/ml) and *Cs*TPs (40 μg/ml) pretreated with 0.1, 1 or 10 μg/ml PZQ (ETV+*Cs*TP+PZQ), for 12, 24 or 48 h at 37 °C in 5% CO_2_ in an incubator. The concentrations of ETV, *Cs*TPs and BSA used were based on our published study [16]. Finally, HBsAg, HBeAg and HBV DNA copies in the supernatants from each group were analysed using ECLI and Q-PCR. All assays were performed in triplicate and repeated twice.

### Statistical analysis

All data are presented as the mean ± standard error (SE) or the median and range. Data analyses were carried out using GraphPad Prism software 7.0 (GraphPad Software, San Diego, CA, USA). One-sided paired Student’s t-test was used. The Kruskal‒Wallis rank test or one-way analysis of variance test was conducted for comparisons with more than two groups. *P*-values < 0.05 were considered to be statistically significant.

## Results

### Clinical characteristics of the study groups

Patients in both the *C. sinensis* group and the HBV group were found to have higher serum levels of ALT, AST, HA and PCIII than those in the HC group (*P* < 0.05, respectively). Serum levels of ALT, AST, TB and HA were significantly higher in the coinfection group than in both the HBV group and the *C. sinensis* group (*P* < 0.05). There was no difference in the number of HBV DNA copies or PCIII, LN and COLIV levels between the coinfection group and the HBV group (*P* > 0.05). The clinical data are shown in detail in Table 1.

**Table 1:** Clinical characteristics of the enrolled population

Clinical characteristics	HC group (*n* = 29)	HBV group (*n* = 26)	*C. sinensis* group (*n* = 24)	Coinfected group (*n* = 22)
Age (years)	44.86 ± 7.46	42.59 ± 12.21	42.42 ± 9.11	40.73 ± 10.49
Gender (male/female)	26/3	23/3	22/2	20/2
HBV DNA (Log copies/ml)	Negative	4.61 ± 1.91	Negative	5.50 ± 1.70
ALT (U/l)	22.00 (9–73)	62.50 (20–665)***#	43.50 (3–615)*###	233.50 (14–733)***
AST (U/l)	22.00 (16–45)	41.50 (21–401)***#	31.00 (18–191)*###	148.50 (21–382)***
TB (μmol/l)	11.00 (2.80–32.64)	16.30 (6.80–418.00)#	13.27 (5.00–472.10)##	51.15 (8.90–626.50)***
HA (ng/ml)	45.17 (30.00–94.85)	77.93 (30.00–255.35)**#	67.88 (30.00–1000.00)*#	131.20 (30.00–1000.00)***
PCIII (ng/ml)	7.37 (5.06–10.86)	11.00 (4.55–26.11)*	10.59 (1.50–32.39)**	7.45 (1.50–31.06)
LN (ng/ml)	44.00 (16.23–83.84)	71.49 (15.00–161.81)	40.86 (15.00–189.52)	38.95 (15.00–327.13)
COLIV (ng/ml)	34.10 (15.02–86.39)	52.74 (21.62–1000.00)	26.90 (15.00–1000.00)	72.37 (15.00–671.37)

Values in table are shown as the mean ± standard error (SE) or as the median with the range in parentheses, with the exception of Gender

*ALT* Alanine aminotransferase, *AST* aspartate aminotransferase, *COL IV* type IV collagen, *HA* hyaluronic acid, *HBV* hepatitis B virus, *LN* laminin, *PCIII* type III procollagen, *TB* total bilirubin

Kruskal–Wallis rank test was used for comparison among multiple groups: *P < 0.05, **P < 0.01, ***P < 0.001, vs healthy control (HC) group; #*P* < 0.05, ##*P* < 0.01, ###*P* < 0.001, vs co-infected group

### Proliferation assay of LX-2 cells

**Fig. 1:**
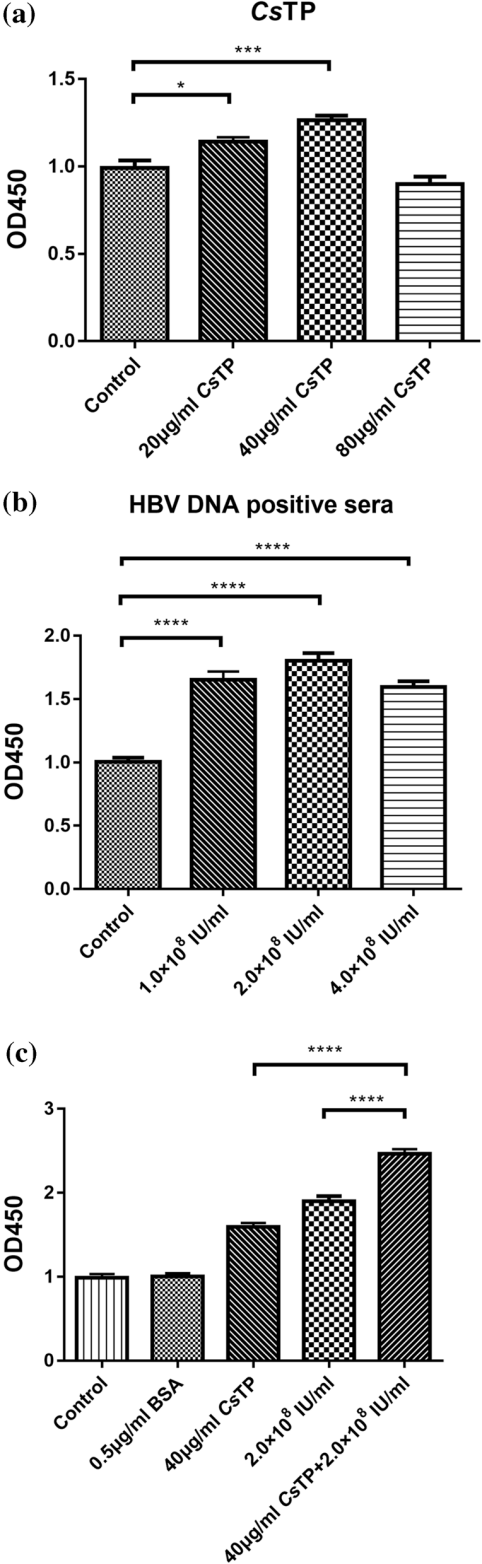
Cell proliferation of human hepatic stellate cell line LX-2 determined using the CCK-8 assay. LX-2 cells were cultured with different concentrations of *Cs*TPs (**a**), HBV DNA-positive sera (**b**) or a mixture of *Cs*TPs and HBV DNA-positive sera (**c**) for 24 h. Cells treated with phosphate-buffered saline or 0.5 µg/ml BSA were used as controls. Data are presented as mean values ± standard errors (SEs). Statistical significance was analysed by one-sided paired Student’s t-test, and asterisks indicate significance vs the Control group at *P < 0.05, ***P < 0.001 BSA, Bovine serum albumin; *Cs*TPs total proteins extracted from adult *Clonorchis sinensis*; HBV, hepatitis B virus

LX-2 cells were cultured with different concentrations (0, 20, 40 or 80 μg/ml) of *Cs*TPs or with HBV-positive sera with different HBV DNA copy numbers (0, 1.0 × 10^8^, 2.0 × 10^8^ or 4.0 × 10^8^ HBV DNA IU/ml, respectively). The highest OD_450_ value of LX-2 cells was recorded following the incubation of the cells with 40 μg/ml *Cs*TPs or with serum containing 2 × 10^8^ IU/ml HBV DNA, according to the CCK-8 assay, suggesting the strongest proliferation (Fig. 1). In addition, no significant difference in cell proliferation was observed in the 0.5 μg/ml BSA-treated group. Subsequently, 40 μg/ml *Cs*TPs and serum containing 2 × 10^8^ IU/ml HBV DNA were employed to investigate the coeffects of *Cs*TPs and HBV on LX-2 cells. Cells incubated with 0.5 μg/ml BSA were used as a control.

### mRNA levels of molecules related to hepatic fibrosis in treated LX-2 cells

**Fig. 2:**
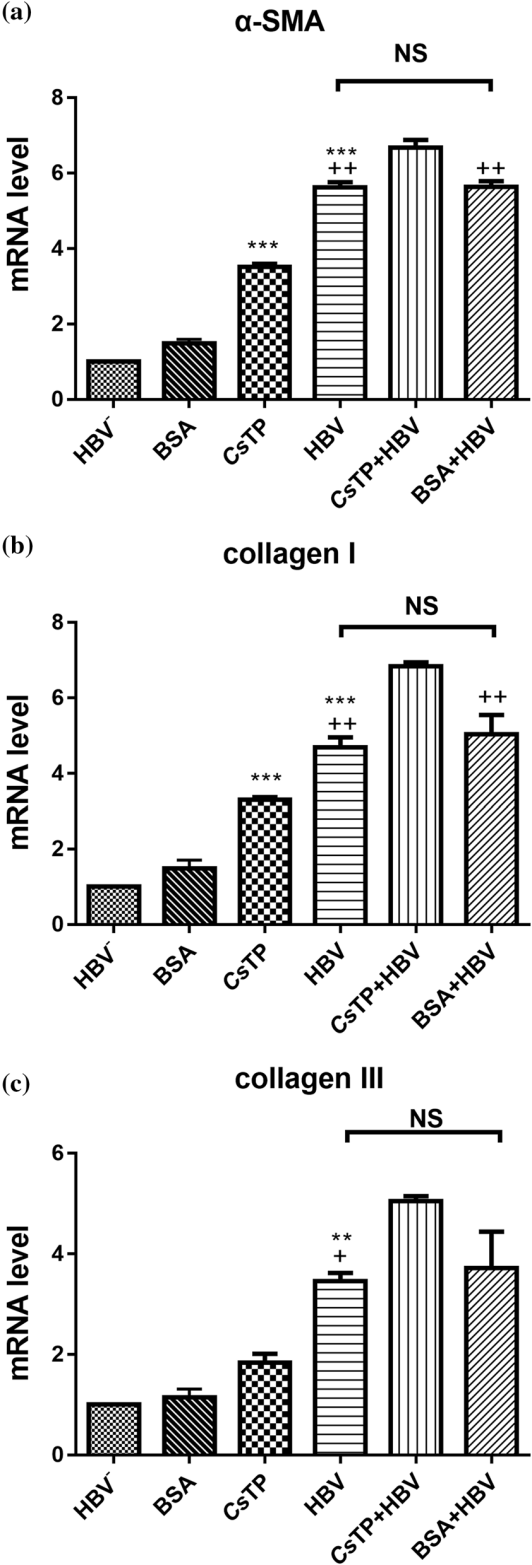
mRNA levels of α-SMA (**a**), collagen I (**b**) and collagen III (**c**) in LX-2 cells by Q-PCR. LX-2 cells were stimulated by *Cs*TPs, HBV positive serum, or a mixture of *Cs*TPs and HBV positive serum (*Cs*TP+HBV). LX-2 cells treated with HBV negative sera, BSA, or a mixture of BSA and HBV positive serum (BSA+HBV) were used as control groups. Specific primers were used to amplify the transcripts of related molecules from total RNAs of the LX-2 cells from each group by Q-PCR. mRNA levels were normalized relative to the β-actin expression. Data were shown as the mean values ± standard errors. Analysis by one-way ANOVA test indicated significant differences among the different groups (*P < 0.05, **P < 0.01, ***P < 0.001 vs the control group. ^+^*P* < 0.05, ^++^*P* < 0.01, ^+++^*P* < 0.001 vs the *Cs*TP+HBV group)

The mRNA levels of α-SMA and types I and III collagen were significantly higher in both the *Cs*TP group and the HBV group than in the HBV-negative group and the BSA control groups (*P* < 0.05). Transcripts of α-SMA and types I and III collagen were significantly elevated in the CsTP+HBV group compared with the HBV group (*P* < 0.05). The mRNA levels of α-SMA and types I and III collagen were higher in the *Cs*TP+HBV group than in the BSA+HBV group, with the first two factors showing significant increases (*P* < 0.01) (Fig. 2).

### mRNA levels of related inflammatory cytokines in treated LX-2 cells

**Fig. 3:**
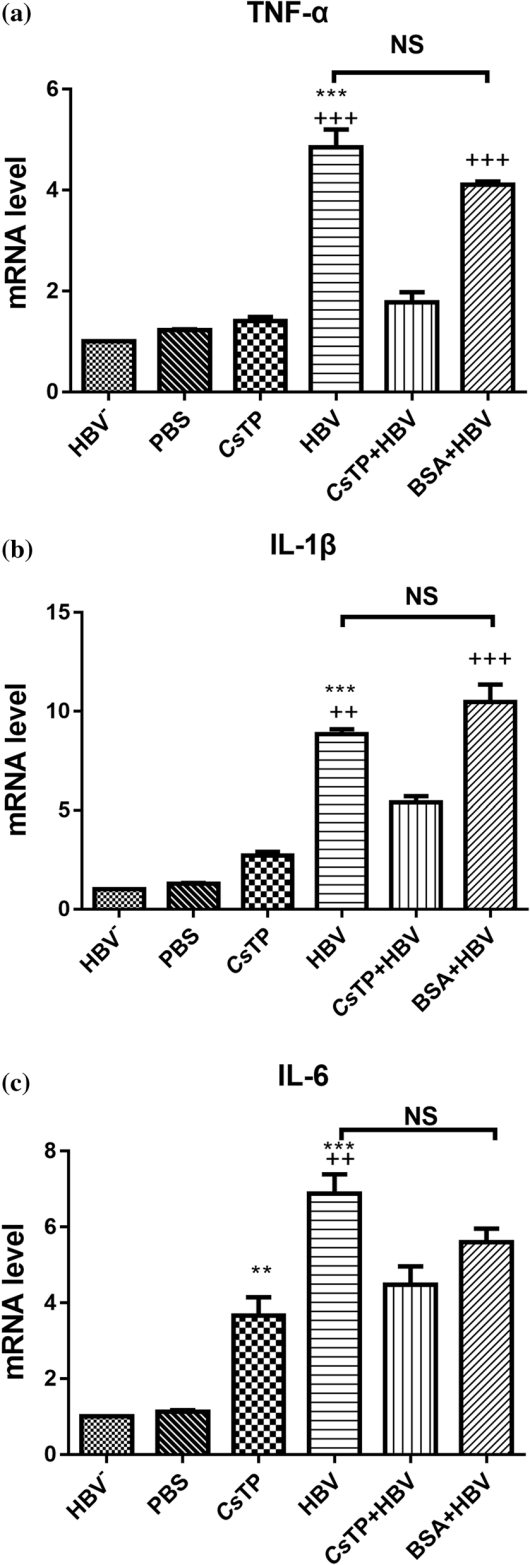
mRNA levels of TNF-α (**a**), IL-1β (**b**) and IL-6 (**c**) in LX-2 cells by Q-PCR. LX-2 cells were incubated with *Cs*TPs, HBV positive serum, or a mixture of *Cs*TPs and HBV positive serum (*Cs*TP+HBV). Cells treated with HBV negative sera, PBS, or a mixture of BSA and HBV positive serum (BSA+HBV) were used as controls. Specific primers were used to amplify the transcripts of related molecules from total RNAs of LX-2 cells in each group by Q-PCR. mRNA levels were normalized relative to β-actin expression. Data were shown as the mean values ± standard errors. Statistical significance was analyzed by one-way ANOVA test (*P < 0.05, **P < 0.01, ***P < 0.001 vs control group; ^+^*P* < 0.05, ^++^*P* < 0.01, ^+++^*P* < 0.001 vs the *Cs*TP+HBV group)

Compared with the levels of TNF-α and IL-1β mRNA in the blank controls (cells incubated with HBV-negative sera or PBS), those in the HBV group and the BSA+HBV group were clearly elevated (*P* < 0.01), but not in the *Cs*TP group (*P* > 0.05). The levels of IL-6 transcripts in all groups were substantially increased compared with those in the blank control group. Compared with the *Cs*TP+HBV group, the HBV group had strongly increased levels of TNF-α, IL-1β and IL-6 mRNA (*P* < 0.01). In addition, the transcription levels of TNF-α and IL-1β in the BSA+HBV group were significantly elevated compared with those in the *Cs*TP+HBV group (*P* < 0.001). There was no significant difference in the levels of TNF-α, IL-1β and IL-6 mRNA between the HBV group and the BSA+HBV group (Fig. 3).

### HBsAg, HBeAg and HBV DNA copy levels of treated HepG2.2.15 cells

**Fig. 4:**
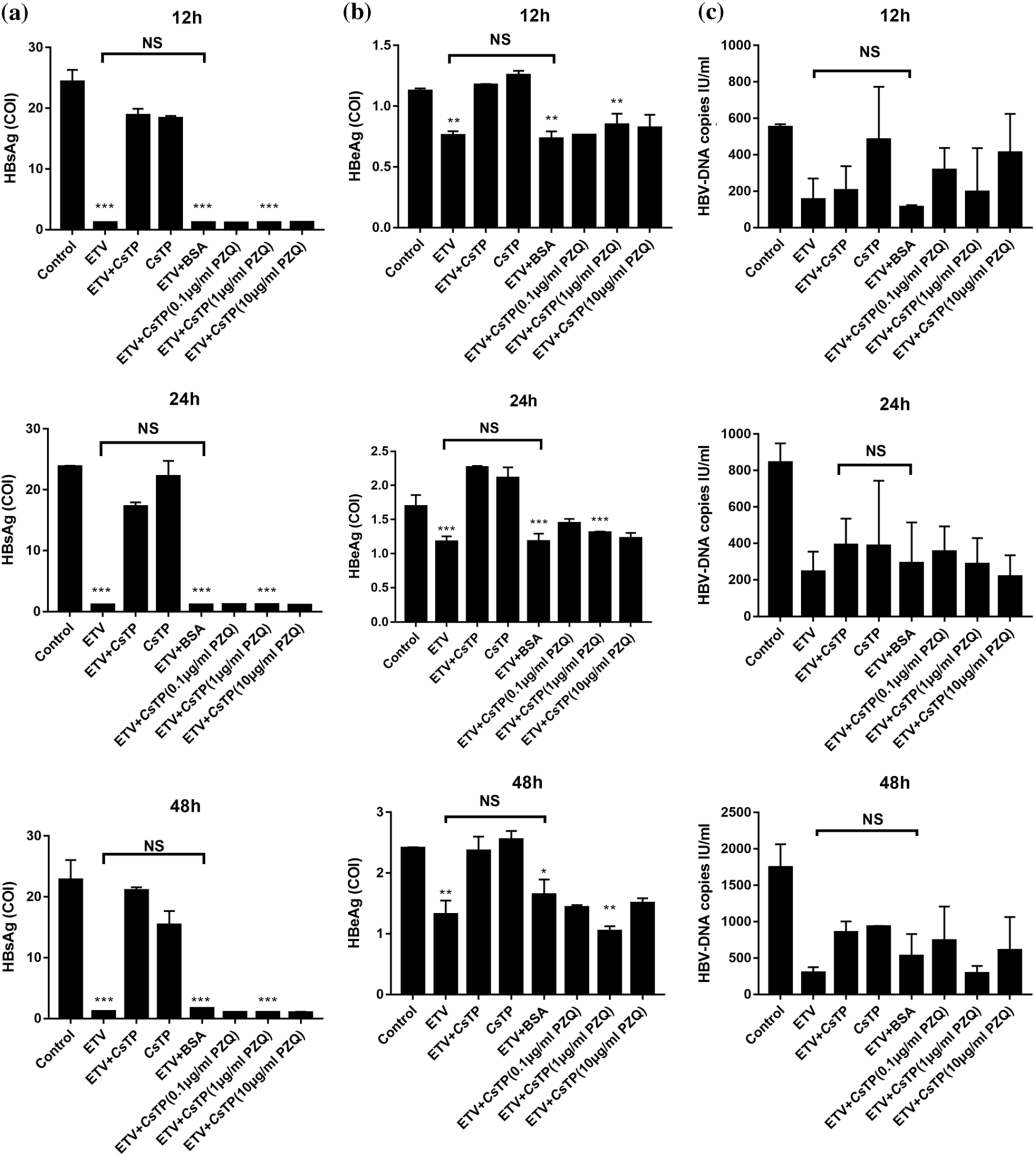
Detection of HBsAg, HBeAg and HBV DNA copies in the supernatant of HepG2.2.15 cells. HepG2.2.15 cells were incubated with ETV, *Cs*TPs, a mixture of ETV and *Cs*TPs (ETV+*Cs*TP), a mixture of ETV and BSA (ETV+BSA) or a mixture of ETV and *Cs*TPs, respectively pretreated with 0.1, 1 or 10 μg/ml PZQ (ETV+CsTP[PZQ]). In the supernatants of HepG2.2.15 cells, levels of HBsAg (**a**) and HBeAg (**b**) were detected by electrochemiluminescence immunoassay, and HBV DNA copies (**c**) were detected by Q-PCR, at the indicated time points (12, 24 and 48 h). Data were presented as mean values ± SEs. Analysis by one-way ANOVA test indicated significant differences among the different groups at ***P < 0.001 vs the ETV+*Cs*TP group. BeAg, HBV e-antigen; HBsAg, HBV surface antigen; ETV, entecavir; PZQ, praziquantel

After 12, 24 and 48 h of incubation, HBsAg and HBeAg levels were significantly higher in the culture supernatants of HepG2.2.15 cells in the ETV+CsTP group than in those of the ETV group or the ETV+BSA group (*P* < 0.05). Moreover, HBsAg and HBeAg levels were predominantly lower in all ETV+CsTP+PZQ groups than in the ETV+CsTP group (*P* < 0.01). There was no significant difference between the ETV group and the ETV+BSA group (Fig. 4a, b).

HBV DNA copies in the supernatant of cells in the ETV+CsTP group increased slightly compared with those in the ETV group or the ETV+CsTP + PZQ (1 μg/ml) group at 12, 24 and 48 h after administration, but there was no significant difference among the three groups. Moreover, there was no significant difference in HBV DNA copies between the ETV group and the ETV+BSA group (Fig. 4c).

## Discussion

In the present study, our clinical data indicated that the levels of ALT, AST, TB and HA were significantly higher in patients with coinfection than in HBV-infected patients. In the cell models, the results showed that transcripts of α-SMA and types I and III collagen were significantly elevated in the *Cs*TP+HBV group compared with the HBV group. The levels of TNF-α, IL-1β and IL-6 mRNA in the *Cs*TP + HBV group were clearly lower than those in the HBV group. HBsAg and HBeAg levels in the ETV+*Cs*TP group were higher than those in the ETV group and in the ETV+*Cs*TP+PZQ group.

The serum marker enzymes ALT and AST are known to be important diagnostic factors for hepatic diseases, and their increased activity in the circulation reflects damage and leakage of hepatocytes. A high TB level is a sign of liver disease. Analysis of the ALT, AST and TB levels in sera from patients demonstrated that *C. sinensis* and HBV coinfection could weaken liver function more than HBV infection alone, which further confirmed our previous finding [14]. Our analysis showed that the levels of HA and COLIV were higher in the coinfection group than in the HBV group, but HA increased significantly. The serum indices of HA, LN, PC III and COL IV can effectively reflect the condition of liver injury and fibrosis, and they are commonly used as clinical indicators to reflect liver fibrosis [21]. HA is a component of the extracellular matrix (ECM) of the liver [22], and serum HA levels correlate well with the severity of liver dysfunction in patients with chronic liver disease [23, 24]. It has been reported that almost 90% of circulating HA is degraded in the liver by Ito cells and sinusoidal cells and, therefore, hepatic dysfunction deteriorates HA clearance [25]. It has been repeatedly found that HA is the most sensitive marker among the four serum indicators of liver fibrosis [26–28]. We found that the HA level was significantly higher in the coinfection group than in the HBV group or the *C. sinensis* group. These results suggested that coinfection might aggravate the progression of liver fibrosis.

Hepatic stellate cells (HSCs) localize to the perisinusoidal space between hepatocytes and sinusoidal endothelial cells. In response to liver injury, inflammatory mediators promote HSC activation. Once activated, HSCs become proliferative, subsequently differentiate into myofibroblasts and then accumulate ECM that drives the fibrogenic process [29]. HSCs play a central role in liver fibrosis by generating fibrotic mediators [30, 31]. When quiescent HSCs were activated by various liver stimuli, a new myofibroblastic phenotype with increased expression of ECM proteins, including collagen I and collagen III and α-SMA, was obtained [32, 33]. Activated human HSCs can produce inflammatory cytokines, such as TNF-α, IL-1β and IL-6. These inflammatory cytokines are potent pleiotropic cytokines that can trigger multiple signalling pathways involved in inflammation, proliferation and apoptosis [34–36]. Moreover, they are essential for liver inflammation, and sustained liver inflammation leads to liver fibrosis. Therefore, in the current study, the human HSC cell line LX-2 [37] was employed to investigate the effects of coinfection on liver fibrosis in vitro.

It has been documented that some antigens from parasites can play important roles in proinflammatory or anti-inflammatory activities [38–40]. We previously revealed that *Cs*TPs could promote the production of Th2 cytokines, including IL-13 and IL-4, both in vitro and in vivo, and ultimately resulted in the development of liver fibrosis [16]. During the parasitic period, proteins from the tegument of adult worms, eggs and excretory-secretory proteins excluded from worm bodies and proteins from the decomposition of dead worms all participate in the interaction between the worms and the host, so *Cs*TPs were employed to carry out the investigation in vitro.

We found that the mRNA levels of α-SMA, collagen I and collagen III in the LX-2 cells stimulated by either *Cs*TPs or HBV-positive sera could be significantly increased compared with those in the control groups (HBV-negative group and BSA group). It has been documented that the HBV X protein and HBsAg can directly upregulate the expression of α-SMA and collagen I [41, 42]. We previously found that *Cs*TPs could also directly stimulate the proliferation of LX-2 cells and activate them [43, 44]. This change might result in the levels of α-SMA and types I and III collagen being notably elevated in response to mixtures of *Cs*TPs and HBV-positive sera compared with those in the HBV group or BSA+HBV group. However, there was no difference between the HBV group and the BSA+HBV group. When quiescent HSCs were activated by various liver stimuli, a new myofibroblastic phenotype with increased ECM protein expression, including the expression of types I and III collagen and α-SMA, was obtained [33, 45]. Our results demonstrated that molecules from either HBV or *C. sinensis* could activate HSCs and improve the generation of ECM components. Moreover, the activation effect of HSCs induced by a mixture of HBV and *Cs*TPs was more conspicuous compared to that of the other groups. Combined with the finding that HA levels in sera from the HBV and *C. sinensis* coinfection group were significantly higher, this result suggested that coinfection could aggravate the development of hepatic fibrosis.

During activation, HSCs lose the ability to express hepatocyte growth factor but they do produce several other growth factors, cytokines and chemokines, such as IL-6, TNF-α, transforming growth factor beta (TGF-β), monocyte chemotactic protein 1 and cytokine-induced neutrophil chemoattractant, that participate in the initiation and progression of liver disease [46]. Incubation of quiescent HSCs with lipopolysaccharide (LPS) increases the synthesis of the proinflammatory cytokines IL-6, TNF-α and IL-1β [47, 48]. In our study, the levels of TNF-α, IL-1β and IL-6 mRNA in LX-2 cells from the HBV group increased substantially in comparison with those from the blank control groups (LX-2 cells treated with HBV-negative sera or BSA). In the acute phase of an infection, plasma levels of the inflammatory cytokines TNF-α, IL-1β and IL-6 increase, followed by enhanced secretion of acute-phase proteins that contribute to antimicrobial defence. Based on this result, we suggest that HBV proteins could act as stimuli and clearly promote the generation of proinflammatory cytokines in LX-2 cells. Our previous study showed that Th2-type cytokines increased in the liver tissue of *Cs*TP-immunized mice [16]. Th2-type cytokines have more of an anti-inflammatory response, and this increase might explain why there was no significant difference in the transcriptional levels of TNF-α and IL-1β between the *Cs*TP group and the blank group and why the levels of proinflammatory cytokines in the *Cs*TP+HBV group were significantly lower than those in the HBV group. IL-6 is the major inducer of hepatic acute phase proteins, and its synthesis and secretion are induced during inflammatory conditions, such as following the stimulation of Toll-like receptor (TLR)-4 by LPS or upon stimulation of cells by IL-1 or TNF-α [49]. Our results showed that the transcriptional level of IL-6 in the *Cs*TP group was significantly elevated in comparison with that in the blank control group. Combined with our previous finding [16], these results suggested that the increase in IL-6 was mainly due to *Cs*TPs acting as pathogen-associated molecular patterns rather than being induced by IL-1 or TNF-α.

IL-6 can directly induce the transition of HSCs towards myofibroblast-like cells and promote collagen secretion in HSCs [50]. During liver fibrosis, HSCs are a cellular source of IL-6 [51], which may might partly contribute to the elevation of α-SMA and type I and III collagens in HBV-positive sera and/or *Cs*TP-treated LX-2 cells. Our results showed that the levels of proinflammatory cytokines in the *Cs*TP+HBV group were lower than those in the HBV group, while the levels of liver fibrosis-related molecules in the *Cs*TP+HBV group were higher than those in the HBV group. Excessive proinflammatory responses can lead to uncontrolled tissue damage, while Th2 responses counteract inflammatory cytokine-mediated microbicidal action. Hepatic fibrosis is the wound-healing response of the liver to many causes of chronic injury. This result suggested that *Cs*TPs might weaken the inflammatory response and perpetuate chronic inflammation in the liver so that coinfection could exacerbate the development of hepatic fibrosis. *Clonorchis sinensis* infection might lead to the chronicity of HBV infection. This finding should be further confirmed in the future.

We previously found that compared to sera of coinfected patients treated with only one cycle of ETV, sera of coinfected patients treated with a combined antiviral drug (ETV) and anti-*C. sinensis* drug (PZQ) (combination therapy) had lower levels of HBV DNA copies and TB levels [52]. In the present study, a HepG2.2.15 cell model was adopted to explore the efficacy of different treatment strategies in vitro. HepG2.2.15 cells stably transfected with the HBV genome have been frequently used in studies of HBV infection and drug treatment [53]. ETV, an inhibitor of HBV DNA polymerase, is an antiviral medication used in the treatment of HBV infection. ETV treatment has been approved as a safe and effective nucleoside therapy in patients with HBeAg-positive chronic hepatitis [54]. In the cytoplasm, HBsAg connects with the HBV genome and creates a complete viral particle. Decreased HBsAg synthesis leads to the intracellular inhibition of virus production [55]. HBeAg is a serologic marker associated with high levels of viral replication and infectivity, and HBV DNA is a quantitative virologic marker reflecting HBV replication level. In vitro, our results showed that the levels of HBsAg and HBeAg were predominantly elevated in the ETV+*Cs*TP group compared with the ETV group. The level of HBV DNA copies was higher in the ETV+*Cs*TP group than in the ETV group, but the difference was not statistically significant. This result demonstrated that *Cs*TPs could weaken the effects of ETV. A possible dissociation between HBeAg and HBV DNA levels has been detected previously [56]. In addition, the immune response is necessary for the inhibition of HBV DNA replication [57, 58]. Therefore, conspicuous changes in HBV DNA copies could not be observed in the cell model, possibly explaining why there was no significant difference in HBV DNA copies between the ETV+*Cs*TP group and the ETV+*Cs*TP+PZQ group. The levels of HBsAg and HBeAg were significantly lower in the ETV+*Cs*TP+PZQ group than in the ETV+*Cs*TP group at 12, 24 and 48 h after administration. PZQ is the preferred drug against *C. sinensis* and other parasitic flatworm infections [59]. It has been documented that PZQ can inhibit lactate dehydrogenase activity of *Cotylophoron cotylophorum* and *Taenia asiatica *in vitro [60, 61]. It has also recently been documented that PZQ activates a transient receptor potential melastatin ion channel (TRPM_PZQ_) in schistosomes by engaging a hydrophobic ligand binding pocket within the voltage sensor-like domain of the channel to cause calcium entry and worm paralysis [62]. The homologues in *C. sinensis* might be influenced by PZQ, which successively reversed the effects of *Cs*TPs on the expression of HBsAg and HBeAg in the supernatant of HepG2.2.15 cells. These results also suggested that *Cs*TPs might directly affect HepG2.2.15 cells but does not depend on the immune response. The involved mechanism should be further investigated.

## Conclusions

Collectively, the clinical liver indices, including ALT, AST, TB and HA, were significantly higher in patients with coinfection than in HBV-infected patients. Moreover, α-SMA and types I and III collagen were dominantly elevated in the LX-2 cell model treated with *Cs*TPs and HBV-positive sera. Taken together, these findings confirm that *C. sinensis* and HBV coinfection could aggravate the progression of liver fibrosis. The present study showed that the levels of TNF-α, IL-1β and IL-6 mRNA were clearly lower in the HepG2.2.15 cell model incubated with *Cs*TPs and HBV-positive sera. Combined with the observation coinfection could exacerbate the imbalance of Th1/Th2 cytokine in our previous study, these results suggest that coinfection might promote hepatic fibrosis by perpetuating chronic liver inflammation. Moreover, *Cs*TPs could weaken the effects of ETV on HepG2.2.15 cell. Our study will broaden our understanding of the relationship between *C. sinensis* and HBV. Therefore, the importance of *C. sinensis* infection in hepatitis B patients should be realized. Our research also provids useful information for physicians to carry out individualized treatment in hepatitis B patients in endemic areas of *C. sinensis*.

## Supplementary Information

**Additional file 1**: **Table S1**. Primer sequences for Q-PCR

## Data Availability

The data supporting the conclusions of this article are included within the article.

## Abbreviations

ALT: Alanine aminotransaminase
AST: Aspartate aminotransferase
*Cs*TPs: Total proteins extracted from adult *C. sinensis*
COLIV: Type IV collagen
ECLI: Electrochemiluminescence immunoassay
ECM: Extracellular matrix
ETV: Entecavir
HA: Hyaluronic acid
HBeAg: Hepatitis B e-antigen
HBsAg: Hepatitis B surface antigen
HBV: Hepatitis B virus
HSCs: Hepatic stellate cells
IL-1β: Interleukin-1β
LN: Laminin
LPS: Lipopolysaccharide
PBS: Phosphate-buffered saline
PCIII: Type III procollagen
PZQ: Praziquantel
Q-PCR: Quantitative real-time PCR
α-SMA: Alpha-smooth muscle actin
TB: Total bilirubin
TNF-α: Tumour necrosis factor-α
TLR: Toll-like receptor
